# Integrative Omic Profiling Reveals Unique Hypoxia Induced Signatures in Gastric Cancer Associated Myofibroblasts

**DOI:** 10.3390/cancers11020263

**Published:** 2019-02-23

**Authors:** Hanna Najgebauer, Andrew F. Jarnuczak, Andrea Varro, Christopher M. Sanderson

**Affiliations:** 1 Department of Cellular and Molecular Physiology, University of Liverpool, Crown Street, Liverpool L69 3BX, UK; A.Varro@liverpool.ac.uk (A.V.); C.Sanderson@liverpool.ac.uk (C.M.S.); 2 European Molecular Biology Laboratory, European Bioinformatics Institute (EMBL-EBI), Wellcome Trust Genome Campus, Hinxton, Cambridge CB10 1SD, UK; jarnuczak@ebi.ac.uk

**Keywords:** cancer-associated myofibroblasts, hypoxia, tumour microenvironment, gene expression, secretome, transcriptomics, proteomics, gastric cancer, omics

## Abstract

Although hypoxia is known to contribute to several aspects of tumour progression, relatively little is known about the effects of hypoxia on cancer-associated myofibroblasts (CAMs), or the consequences that conditional changes in CAM function may have on tumour development and metastasis. To investigate this issue in the context of gastric cancer, a comparative multiomic analysis was performed on populations of patient-derived myofibroblasts, cultured under normoxic or hypoxic conditions. Data from this study reveal a novel set of CAM-specific hypoxia-induced changes in gene expression and secreted proteins. Significantly, these signatures are not observed in either patient matched adjacent tissue myofibroblasts (ATMs) or non-cancer associated normal tissue myofibroblasts (NTMs). Functional characterisation of different myofibroblast populations shows that hypoxia-induced changes in gene expression not only enhance the ability of CAMs to induce cancer cell migration, but also confer pro-tumorigenic (CAM-like) properties in NTMs. This study provides the first global mechanistic insight into the molecular changes that contribute to hypoxia-induced pro-tumorigenic changes in gastric stromal myofibroblasts.

## 1. Introduction

Gastric cancer remains a leading cause of cancer death worldwide [1]. It is now clear that many tumours develop as a result of complex reciprocal interactions between cancer cells and neighbouring cells within the tumour microenvironment. These include fibroblasts, myofibroblasts, endothelial cells, and immune cells, which together contribute to the formation of a specialised extracellular matrix, and soluble paracrine factors [2,3] which support the growth and metastasis of gastric tumours. In contrast to many other tissues, ‘activated’ myofibroblasts are well represented in normal gastric tissue, where they are closely allied to the epithelium. However, in gastric cancers, resident myofibroblasts acquire additional tumour promoting functions [4]. In particular, gastric cancer-associated myofibroblasts (CAMs) secrete factors that increase the migration, invasion, and proliferation of cancer cells, when compared to either adjacent tissue myofibroblasts (ATMs), or normal tissue myofibroblasts (NTMs) [5]. This tumour-promoting phenotype is maintained, at least in part, due to a robust CAM-specific DNA methylation signature [6]. Analysis of gastric CAM conditioned media confirmed that these cells exhibit distinct patterns of protein secretion, compared to tissue matched ATMs. Further, a direct correlation was observed between decreased levels of extracellular matrix adaptor proteins, such as transforming growth factor β-induced gene-h3 (TGFβig-h3) in the secretome of gastric CAMs and the occurrence of lymph node metastasis, worse prognosis, and shorter patient survival times [5].

Significantly, a number of other factors that distinguish gastric CAMs from normal non-cancer associated myofibroblasts have been reported. One such factor is galectin-1, which shows increased expression in CAMs and was shown to enhance gastric cancer cell migration and invasion by upregulating integrin-β1 expression [7]. In addition, CAM-derived galectin-1 is also known to promote angiogenesis in gastric cancer [8]. Other examples of CAM specific changes include the upregulation of miR-106b and fibroblast growth factor 9 (FGF-9) [9,10]. FGF-9 promotes the anti-apoptotic and invasive capability of gastric cancer cells [9], whereas upregulated miR-106b is associated with poor patient prognosis, as it enhances gastric cancer cell migration and invasion [10]. Immunohistochemistry and real-time PCR experiments have shown that CAMs frequently accumulate in the stroma of gastric tumours, and the prevalence of CAMs shows a positive correlation with tumour size, depth, and metastasis [11]. Interestingly, studies in scirrhous gastric cancer, which has the worst prognosis among all types of gastric cancer, show that CAMs may also regulate the stemness of cancer stem cells (CSCs) [12], identifying Asporin as a unique CAM-derived secretory protein that promotes the coordinated invasion of both CAMs and cancer cells [13].

As CAMs play a vital role in the progression of gastric cancer, it is critical to define the molecular mechanisms that enable gastric CAMs to facilitate different aspects of tumour progression. This study was designed to compare the molecular modulation of different myofibroblasts populations (CAM, ATM, NTM) in response to hypoxic conditions.

Hypoxia is an important microenvironmental factor which contributes to several aspects of tumour progression, including tumour growth, tissue invasion, and metastatic spread [14,15]. Although significant efforts have been devoted to studying the consequences of hypoxia on cancer cells, relatively little is known about the effects or consequence of hypoxia on the stromal component of the tumour microenvironment. Previously, it was proposed that hypoxia may have a role in modulating the differentiation and activity of stromal cells [16] in such a way that they synergise with hypoxic oxidative stress to enhance tumour aggressiveness in melanomas [17]. In this context, hypoxia-mediated oxidative stress was found to be mandatory for the activation of dermal fibroblasts and secretion of cytokines and other pro-migratory factors, in addition to actively promoting the invasion and chemotaxis of melanoma cells [17]. However, the extent to which exposure to hypoxia may contribute to or change myofibroblast activity in gastric cancer remains largely unexplored. For example, it is not yet known if hypoxia induces a more aggressive CAM phenotype, or if NTMs become more aggressive, or more CAM-like, under hypoxic conditions. Equally, little is known about the induced conditional changes in gene expression that may facilitate pro-tumorigenic changes in myofibroblasts, including changes in the secretome of CAMs or NTMs, which in turn may have the potential to impose changes in cancer cell function.

To address these questions, we employed a combination of cell-based assays and genomic, transcriptomic, and proteomics approaches. Results from this study show that hypoxia enhances CAM-induced cancer cell migration and promotes the acquisition of CAM-like properties in NTMs. Furthermore, our data identify unique gene expression and protein secretion signatures between myofibroblasts purified from different tissue microenvironments, thereby providing a new resource to guide mechanistic investigation into the molecular processes that drive key aspects of tumour stromal communication.

## 2. Results

### 2.1. Hypoxia Enhances CAM-Induced Cancer Cell Migration and Promotes CAM-Like Properties in NTMs

**Figure 1:**
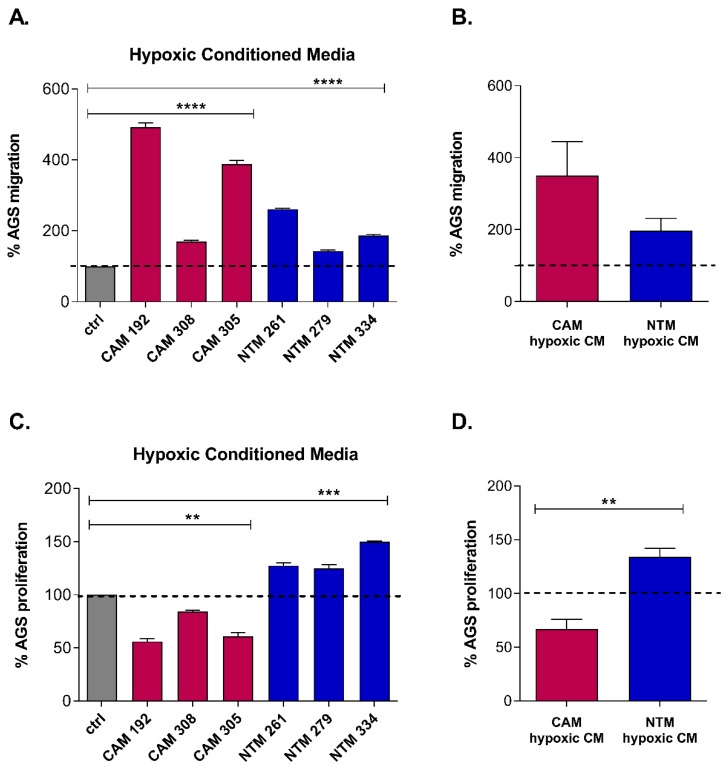
Differential effects of cancer-associated myofibroblasts (CAM) and normal tissue myofibroblasts (NTM) hypoxic conditioned media (CM) on gastric adenocarcinoma cell (AGS) migration and proliferation. (**A,B**). CAM-hypoxic-CM (magenta) and NTM-hypoxic-CM (**blue**) induce AGS cell migration compared to respective CAM or NTM normoxic-control CM (**grey**). (**A**) Individual patient data corrected for AGS basal migration (serum-free media) normalised to respective patient-specific control CM obtained from normoxia (expressed as 100% to enable comparison between effects of CAM and NTM hypoxic conditioned media on AGS migration; for pair-wise comparisons between respective patient-specific ctrl- and hypoxic-CM, see Supplementary Figure S1A); paired *t*-test *p*-value < 0.0001. Error bars represent SD of technical replicates. (**B**) Group mean data (biological replicates) of AGS cell migration in response to hypoxic-CM from CAMs (*n* = 3) and NTMs (*n* = 3); unpaired *t*-test *p*-value > 0.05; Error bars represent SEM. (**C**,**D**). NTM-hypoxic-CM (blue) induces AGS cell proliferation, whereas CAM-hypoxic-CM (magenta) reduces the ability of AGS cells to proliferate compared to respective CAM or NTM control CM (grey) obtained from normoxia. (**C**) Individual patient data corrected for AGS basal proliferation (serum-free media) and normalised to respective patient-specific control CM obtained from normoxia (expressed as 100% to enable comparison between effects of CAM and NTM hypoxic conditioned media on AGS proliferation; for pair-wise comparisons between respective patient-specific ctrl- and hypoxic-CM, see Figure S1B); paired *t*-test *p*-value < 0.005. Error bars represent SEM of technical replicates. (**D**) Group mean data (biological replicates) of AGS cell proliferation in response to hypoxic-CM from CAMs (*n* = 3) and NTMs (*n* = 3); unpaired *t*-test *p*-value < 0.005; Error bars represent SEM.

To assess how hypoxia (1% O_2_) may affect the ability of CAMs and NTMs to induce cancer cell migration and/or proliferation, a series of complementary Boyden chamber migration and EdU cell proliferation assays were performed, in which gastric adenocarcinoma cells (AGS) were exposed to fresh CAM or NTM conditioned media (CM), prepared under hypoxic (hypoxic-CM) or normoxic (ctrl-CM) conditions. Results from these studies show that both CAM- and NTM-derived hypoxic-CM significantly enhanced the migration of gastric cancer cells, in comparison to corresponding ctrl-CM derived from either CAMs or NTMs (Figure 1A, Figure S1A). These data confirm that hypoxia induces the expression and release of pro-migratory factors. Interestingly, group mean data show this effect is on average almost twice as large for CAM-hypoxic-CM, as compared to NTM-hypoxic-CM (Figure 1B). By contrast, hypoxia exerted a reciprocal trend with respect to gastric cancer cell proliferation. Compared to ctrl-CM, CAM-hypoxic-CM consistently reduced gastric cancer cell proliferation, while NTM-hypoxic-CM induced a reproducible increase in cancer cell proliferation (Figure 1C, Figure S1B). On average, exposure to hypoxia conferred a ~40% decrease in CAM-stimulated cancer cell proliferation, in contrast to a ~30% hypoxia-induced increase in NTM-stimulated cancer cell proliferation (Figure 1D). Together, these results suggest that hypoxic conditions may promote some CAM-like properties in gastric NTMs. In particular, exposure to hypoxic conditions would allow non-cancer conditioned NTMs within the surrounding stroma to contribute to hypoxia-enhanced proliferation and spread of metastatic gastric cancer cells. To investigate the molecular mechanisms that confer hypoxia-induced changes in different myofibroblast populations, a comparative global gene expression analysis was performed on patient-matched gastric CAMs and ATMs, and unrelated NTMs.

### 2.2. Hypoxia Confers Differential Changes in Gene Expression in Gastric Myofibroblasts Purified from Different Tissue Microenvironments

**Figure 2:**
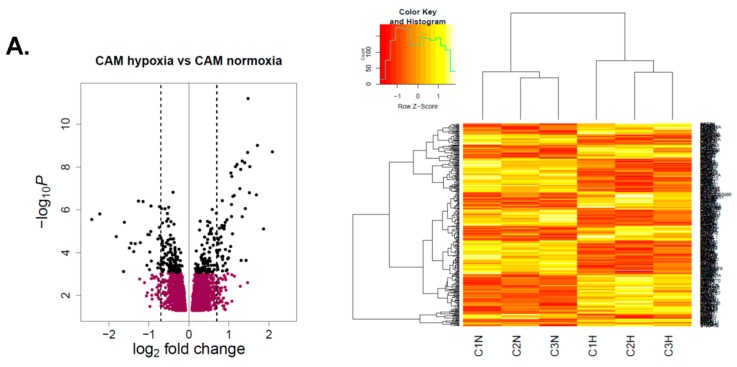

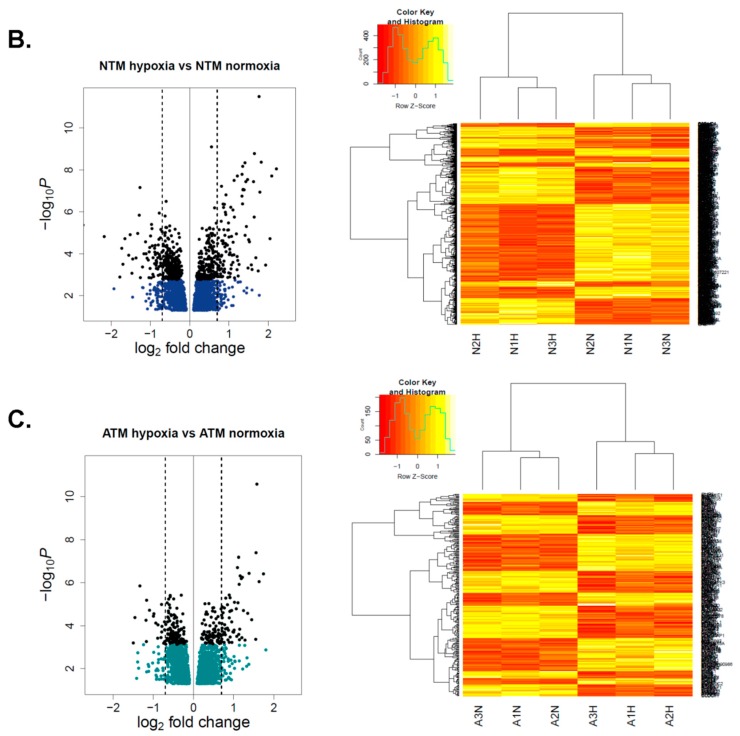
Hypoxia-induced gene expression signatures in gastric myofibroblasts purified from different tissue microenvironments. (**A**) CAM hypoxia vs. CAM normoxia. (**B**) NTM hypoxia vs. NTM normoxia. (**C**) ATM hypoxia vs. ATM normoxia. Left: volcano plots show the distribution of log2 fold changes (*x*-axis) versus -log10 *p*-values (*y*-axis) in respective comparisons. Significantly changing genes (FDR (False discovery rate) *p*-value < 0.05) are highlighted in black. Dashed lines correspond to a 1.6-fold change (log2 fold change of 0.58). Points >0 signify upregulation by hypoxia. Right: Unsupervised hierarchical clustering of the differentially expressed genes (shown in black in volcano plots).

To compare hypoxia-induced gene expression signatures in gastric myofibroblasts purified from different tissue microenvironments, Illumina HumanHT-12v4 Expression BeadChip arrays were performed on a collection of patient-matched CAM and ATM samples and unrelated NTM samples. In each case, myofibroblasts were cultured in parallel under either hypoxic (1% O_2_) or normoxic (21% O_2_) conditions for 72 h. Overall, 13381 genes were consistently expressed in all samples. Of those, 2467 genes were found to be differentially expressed in hypoxic CAMs compared to control CAMs (Figure 2A). In comparison, 2722 genes were found to be significantly altered in hypoxic vs. normoxic NTMs (Figure 2B), and 2561 genes were differentially expressed in hypoxic vs. normoxic ATMs (Figure 2C). Notably, unsupervised hierarchical clustering correctly grouped samples by condition and identified clear clusters of genes, which reflect transcriptome remodelling in response to hypoxia (Figure 2).

#### 2.2.1. Differential Hypoxia-Induced Myofibroblast Phenotypes Are Not Due to Changes in DNA Methylation

**Figure 3:**
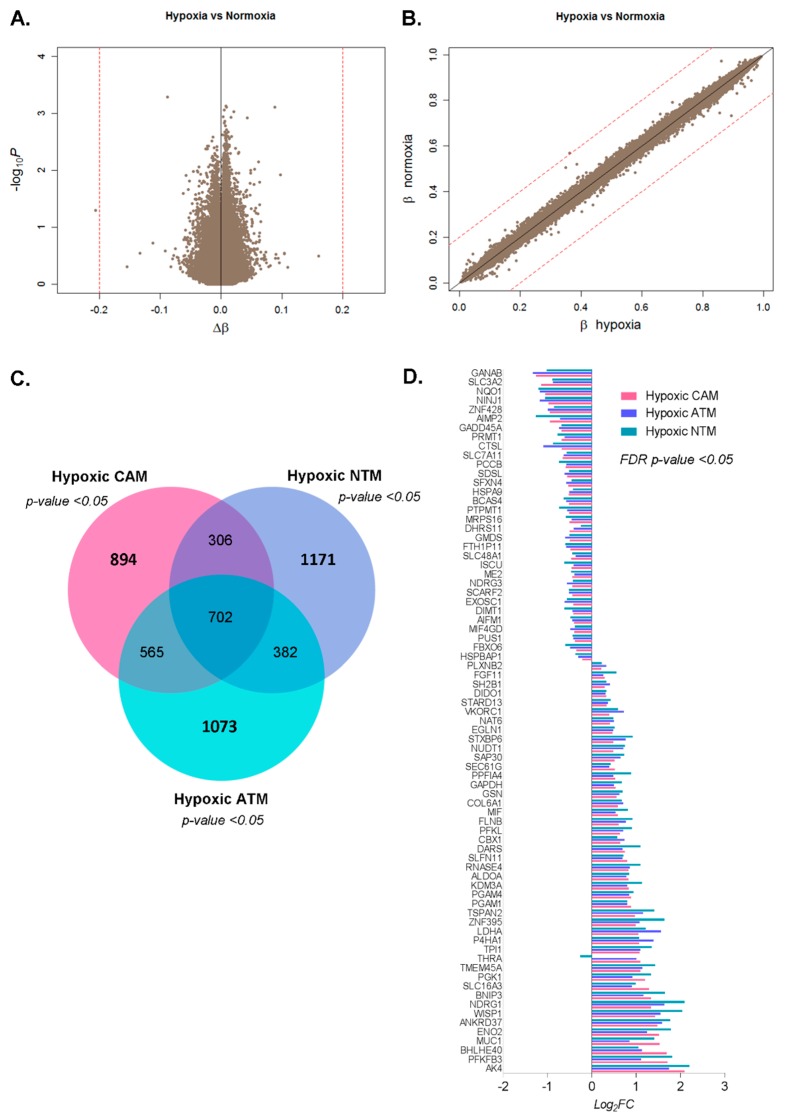
Hypoxia-induced gene expression signatures are not conferred by associated changes in DNA methylation. (**A**,**B**) Comparative global DNA methylation profiles of primary gastric myofibroblasts following exposure to hypoxic or normoxic conditions. (**A**) Volcano plot showing significance versus Δβ values for 424383 methylation probes in the three hypoxia (CAM, ATM, and NTM; *n* = 9) vs. normoxia (CAM, ATM and NTM; *n* = 9) comparisons. (**B**) Scatter plot representing mean β values for normoxic (CAM, ATM and NTM; *n* = 9) and hypoxic (CAM, ATM, and NTM; *n* = 9) myofibroblasts. Red dashed lines represent |Δβ| > 0.2. (**C**,**D**). Comparison of hypoxia-induced gene expression signatures identified in gastric CAMs, ATMs, and NTMs. (**C**) Venn diagram showing the overlap between significantly changed genes (limma *p*-value < 0.05) in response to hypoxia in CAM, ATM, and NTM cells. The common intersection represents a universal hypoxia-induced gene signature. Unique CAM-, ATM-, and NTM- hypoxia-induced gene signatures are highlighted in bold. (**D**) Genes universally changed in the same direction under hypoxia across all three populations of gastric myofibroblasts (CAMs, ATMs, and NTMs, limma *FDR p*-value < 0.05).

To assess whether observed changes in hypoxia-induced gene expression profiles are the result of conditional changes in DNA methylation, parallel Illumina Infinium HumanMethylation450 BeadChip assays were performed on CAMs, patient-matched ATMs, and unrelated NTMs. Comparison of genome–wide DNA methylation profiles between: (i) CAM hypoxia vs. CAM normoxia, (ii) ATM hypoxia vs. ATM normoxia, and (iii) NTM hypoxia vs. NTM normoxia showed a striking similarity in DNA methylation profiles between matched hypoxic and normoxic samples. In all three comparisons, no significant methylation changes were observed. For clarity, this is visualised in a single volcano plot and scatter plot in Figure 3A,B. These data suggest that under the conditions used in this study, hypoxia-imposed changes in gene expression are not conferred by associated changes in DNA methylation patterns.

#### 2.2.2. Hypoxia-Induced Gene Expression Signatures

In general, cells appear to respond to hypoxia via a common HIF-1α mediated mechanism [18,19]; therefore, a subset of common hypoxia-induced gene expression changes may be expected in all populations of myofibroblasts. However, since the net functional response of each population of myofibroblasts (CAMs, ATMs, or NTMs) is different, it is likely that reported variations in epigenetic programming between different myofibroblast populations [6] may confer differences in gene expression, and functional responses to hypoxic conditions. Differential gene expression analyses identified 702 genes that are universally changed under hypoxia in all myofibroblast populations (Figure 3C), including a subset of genes that exhibit conserved directional changes (*p*-value < 0.05) across all myofibroblast types (Figure 3D). Amongst the most upregulated genes are *AK4, PFKFB3, BNIP3*, and *NDRG1*, all of which show around 4-fold increases in expression under hypoxic conditions. In comparison, genes displaying the largest down-regulation in response to hypoxia are *GANAB, SLC3A2, NQO1*, and *AIMP2* (*FC* < −2). To identify signalling pathways that are enriched in the group of 702 universal hypoxia-induced genes, gene ontology (GO) and gene set enrichment (GSEA) analyses were performed. Results from GO term analyses revealed glycolytic processes and metabolic processes to be the most significantly represented (Figure S2). Reassuringly, GSEA analysis detected strong enrichment of gene sets characteristic for hypoxic cells, with ‘hallmark hypoxia’ and ‘hallmark glycolysis’ having the highest normalised enrichment scores. Notably, genes associated with those categories were almost exclusively grouped in the fraction of hypoxia up-regulated genes (Figure S3 and Table S1).

#### 2.2.3. CAMs, ATMs, and NTMs Express Unique Gene Signatures Under Hypoxia

Although common patterns of hypoxia-induced gene expression were observed in all populations of gastric myofibroblasts, functional assays confirmed that each population conferred differential effects on cancer cell migration and proliferation following exposure to hypoxic conditions. To investigate the molecular basis of these functional differences, subsets of unique hypoxia-induced genes were identified in each myofibroblast population. In total, 894 genes were found to be uniquely changed in hypoxic CAMs, 1171 in hypoxic NTMs, and 1073 in hypoxic ATMs (Figure 3C). Each of these distinct conditional response signatures was then subjected to functional enrichment analysis using GO (Table S2), GSEA (Figure S4 and Table S3), and ingenuity pathway analysis (IPA, Figure S5).

Overall, these analyses revealed unique transcriptional responses to hypoxia across different populations of myofibroblasts. Most notably, upregulation of ‘cholesterol biosynthesis’ and ‘fatty acid metabolism’ emerged as a unique feature of hypoxic CAMs, while GSEA showed enrichment for ‘hallmark cholesterol homeostasis’ (Figure S4A). In addition, IPA revealed an upregulation of nearly all components of the ‘superpathway of cholesterol biosynthesis’ (Figure S5). These data strongly suggest that hypoxia enhances production of cholesterol in gastric CAMs, which may contribute to the energy metabolism of adjacent cancer cells. Notably, in patient-matched ATMs, low oxygen concentration uniquely induced the expression of genes involved in the G2/M cell cycle checkpoint, mitotic spindle assembly, and integrin signalling (Figures S4B and S5, Tables S2 and S3). Finally, analysis of the NTM unique gene expression signature showed that these genes simply contribute to signalling pathways related to hypoxia response and xenobiotic metabolism (Figures S4C and S5, Tables S2 and S3). The IPA downstream effects analysis, which facilitates the prediction of functions and processes that are expected to be increased or decreased based on changes in gene expression, also complements these observations (Figure S6).

**Figure 4:**
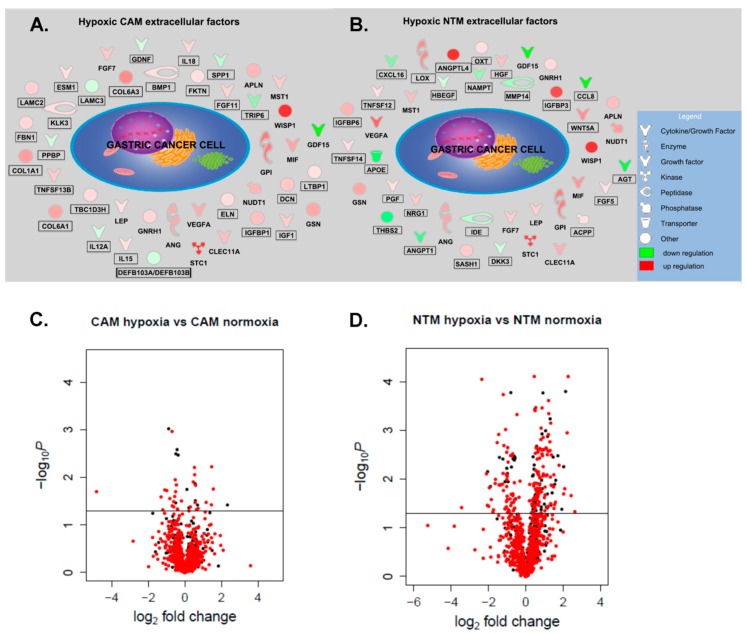
Unique CAMs and NTMs hypoxia signatures may explain differential effects on cancer cell phenotype. (**A**,**B**). Predicted pro-migratory and pro-proliferative factors secreted by hypoxic CAMs and hypoxic NTMs. (**A**). Extracellular molecules expressed by hypoxic CAMs. (**B**). Extracellular molecules expressed by hypoxic NTMs. Molecules displayed in boxes are uniquely expressed in hypoxic CAM or hypoxic NTM. (**C**,**D**). Volcano plots of differentially secreted proteins in (**C**). CAM-hypoxic-CM vs. CAM-ctrl-CM and (**D**). NTM-hypoxic-CM vs. NTM-ctrl-CM comparisons. Proteins highlighted in red were annotated as secreted/extracellular or identified in exosomes based on MetazSecKB, Matrisome, and ExoCarta searches. Proteins above the line (*p*-value < 0.05) are considered to be differentially secreted between the given conditions.

Having identified prominent population-specific differences in myofibroblast responses to hypoxic conditions, it was important to investigate if factors secreted by hypoxic CAMs and NTMs could be predicted, based on changes in gene expression. Therefore, an IPA downstream effects analysis was performed for CAM-hypoxia vs. CAM-normoxia and NTM-hapoxia vs. NTM-normoxia comparisons. The results were then filtered to include only genes that were likely to encode extracellular proteins that may be involved in cell migration and proliferation. This procedure identified 66 pro-migratory and pro-proliferative proteins that are likely to affect the observed cancer cell phenotype in response to CAM- and NTM-hypoxic-CM (Figure 4A,B).

### 2.3. Analysis of Hypoxia-Induced CAM and NTM Secretomes

In order to compare the profile of secreted proteins released by gastric myofibroblasts in response to hypoxia, conditioned media from CAMs and NTMs were profiled using liquid chromatography-mass spectrometry (LC-MS). In total, four conditions were examined: (i) CAM-ctrl-CM; (ii) CAM-hypoxic-CM; (iii) NTM-ctrl-CM; and (iv) NTM-hypoxic-CM. In each case, CM was collected after 72 h exposure to hypoxic (1% O_2_) or normoxic (21% O_2_) conditions. Altogether, 1169 protein groups were identified (*FDR p*-value < 0.01) and quantified across all samples (*n* = 12) using the MaxQuant computational platform. A core of 702 proteins was detected in all CM samples. These were subjected to gene ontology (GO) enrichment analysis using the PANTHER classification system [20] in order to verify their cellular location. The identified proteins were significantly enriched in terms of annotation for extracellular region (*p* = 4.59 × 10^−227^) and extracellular vesicle (*p* = 1.74 × 10^−200^). The other top 10 most significantly enriched GO terms were also associated with extracellular structures, confirming that our experimental strategy worked. In addition, the Metazoa Secretome and Subcellular Proteome Knowledge Base [21], MatrisomeDB [22], and ExoCarta [23] were used to further annotate proteins identified in CAM or NTM secretomes. Overall, 76% were either known or predicted to be extracellular (Figure S7).

### 2.4. Gene Expression Profiles Enable Prediction of Secreted Factors

**Figure 5:**
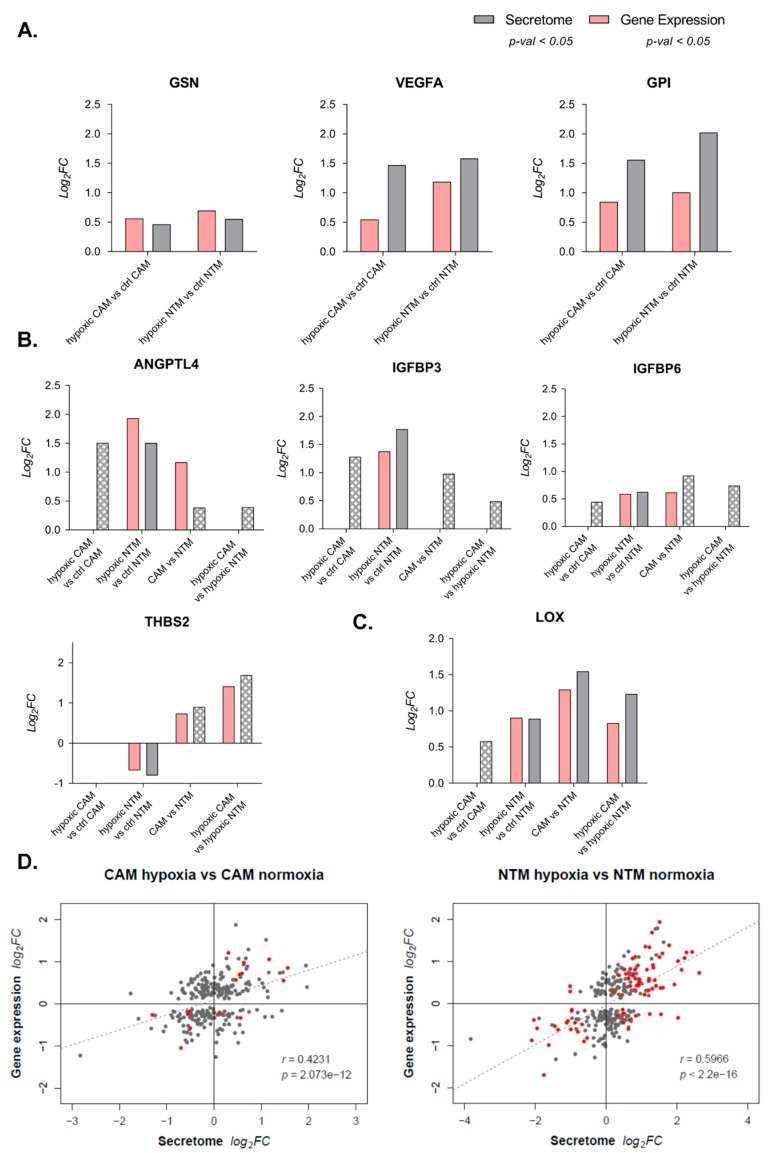
Gene expression profiles enable prediction of secreted factors. (**A**). Differential expression and secretion of gelsolin (GSN), vascular endothelial growth factor A (VEGFA), and glucose-6-phosphate isomerase (GPI) in hypoxic CAMs and hypoxic NTMs compared to respective control-normoxic myofibroblasts; *p*-value < 0.05. (**B**). Differential expression and secretion of angiopoietin-related protein 4 (ANGPTL4), insulin-like growth factor-binding protein 3 (IGFBP3), insulin-like growth factor-binding protein 6 (IGFBP6), and thrombospondin-2 (THBS2) in gastric CAMs and NTMs under hypoxia and normoxia; *p*-value <0.05; checked–proteins detected in respective comparisons but not identified as differentially secreted (*p*-value > 0.05). (**C**). Differential expression and secretion of enzyme lysyl oxidase (LOX) in gastric CAMs and NTMs under hypoxia and normoxia. LOX mRNA transcript was not identified as differentially expressed in hypoxic CAMs compared to control-normoxic CAMs (*p*-value > 0.05), whereas LOX protein (checked) was detected in CAM-hypoxic-CM and CAM-ctrl-CM; however, it was not identified as significant in differential secretome analysis (*p*-value > 0.05). (**D**). Integration of secretome and gene expression data for CAM hypoxia vs. CAM normoxia and NTM hypoxia vs. NTM normoxia. The dashed line represents the best linear fit between the two variables. Grey points indicate that from the mRNA-protein pair, only the transcript was determined to be significantly changing (*p*-value < 0.05), and red points that both protein and transcript were significantly altered (*p*-value < 0.05). *r*–Pearson correlation, *p*–*p*-value indicating the significance of the correlation.

Following an analysis of variance (ANOVA) test, a number of differentially secreted proteins were identified: 43 in the CAM-ctrl-CM vs. CAM-hypoxic-CM comparison (Figure 4C) and 225 in NTM-ctrl-CM vs. NTM-hypoxic-CM comparison (Figure 4D). However, only 14 (5%) of all significantly changed proteins were altered in both hypoxic NTM and CAM secretomes (Figure S8). This reinforced our previous findings that different populations of myofibroblasts display unique molecular signatures in response to environmental changes. Significantly, proteomics analysis also confirmed some conditional secretome signatures that were predicted from the analysis of corresponding gene expression profiles (Figure 4A,B). In particular, gelsolin (GSN), vascular endothelial growth factor A (VEGFA), and glucose-6-phosphate isomerase (GPI) were all verified at protein level to be differentially secreted by both hypoxic CAMs and NTMs (Figure 5A). In addition, hypoxic NTMs increased secretion of lysyl oxidase (LOX), insulin-like growth factor-binding protein 3 (IGFBP3), insulin-like growth factor-binding protein 6 (IGFBP6), and angiopoietin-related protein 4 (ANGPTL4). Furthermore, secretion of those proteins was not significantly altered in hypoxic CAMs (Figure 5B,C), as predicted by IPA. This demonstrates that integration of multiple omics technologies enables validation of results, while also facilitating biologically relevant predictions.

Another finding in concordance with previous gene expression studies relates to the potential biological effects of the secreted proteins. IPA downstream effects analysis indicated that cell death and necrosis may decrease in response to hypoxia-induced changes in CAM secretome composition. Furthermore, proliferation, invasion of tumour cell lines, and angiogenesis were predicted to increase in response to imposed changes in the NTM hypoxia-induced secretome (Figure S9). Notably, proteins secreted by hypoxic NTMs were predicted to be regulated by HIF-1α activation (*z*-score *=* 2.303, *p* = 4.76 × 10^−9^), as revealed by IPA upstream regulator analysis. These hypoxic NTM secreted proteins positively affect angiogenesis, migration of endothelial cells, and proliferation of tumour cell lines (Figure S10). These results provide new insights into the potential mechanisms by which hypoxia may enable stromal myofibroblasts to promote cancer cell proliferation and migration as well as highlighting the molecular components involved in mediating these processes.

### 2.5. Secretion Is Not Strongly Regulated by Transcriptome Changes

Although likely secreted factors can be predicted from gene expression data, the extent to which transcriptomic profiles reflect true patterns of protein expression or secretion remains uncertain. Nevertheless, on a simplistic level, it is possible that in some cases, increased mRNA expression may lead to higher concentrations of the encoded proteins and enhanced levels of secretion, or vice-versa. To identify subsets of proteins which show this correlation, fold changes of differentially expressed genes were compared with corresponding measurements of secreted proteins. For each comparison, a Pearson correlation coefficient (*r*) was calculated and the significance of the relationship was assessed by obtaining the corresponding *p*-value. Overall, a statistically significant correlation was observed between these two variables. In CAMs, the transcriptome showed a weaker yet significant correlation with the secretome (*r* = 0.42), while in NTMs, the co-regulation was substantially higher (*r* = 0.6) (Figure 5D). Furthermore, in both cases, the correlation was positive, indicating that on average, increases in transcript concentration lead to greater protein secretion in response to hypoxia. In addition, the magnitude of the changes was also similar.

## 3. Discussion

Hypoxia is an important microenvironmental factor in the development and metastasis of many solid tumours. During tumour development, both cancer and stromal cells are exposed to hypoxic conditions. This study provides novel evidence that hypoxia induces differences in the functional effects that gastric CAMs and NTMs exert on cancer cell migration and proliferation. In particular, hypoxia significantly increases the ability of CAMs to promote the migration of cancer cells, while also inducing pro-tumorigenic CAM-like properties in gastric NTMs. Genome-wide DNA methylation profiling of hypoxia-treated CAMs and NTMs showed that although CAMs and NTMs have distinctive patterns of DNA methylation [6], these characteristic profiles were not significantly altered by hypoxic conditioning, suggesting that unique transcriptional responses to hypoxia observed among different populations of gastric myofibroblasts (CAMs, ATMs, NTMs) most likely stem from differential retained epigenetic backgrounds. As such, hypoxia, as a single microenvironmental factor, is unlikely to impose CAM-like DNA methylation patterns in normal tissue myofibroblast (NTMs).

Interestingly, upregulation of cholesterol biosynthesis and fatty acid metabolism emerged as a unique feature of hypoxic CAMs, with all components of the “superpathway” for cholesterol biosynthesis showing significant upregulation, indicating that hypoxia may enhance cholesterol production in gastric CAMs. In this context, malignant cells have previously been shown to increase lipolysis in adipocytes, which then secrete fatty acids that are then taken up by neighbouring cancer cells to enhance energy production [24]. Data from this study suggest that under hypoxic conditions, gastric CAMs may serve a similar function. In particular, coupling of energy metabolism between CAMs and cancer cells may be mediated by fatty acid binding proteins, such as FABP1 and FABP3, which were also upregulated in hypoxic CAMs. Finally, upregulation of cholesterol biosynthesis under hypoxia in CAMs may serve as an adaptive mechanism that protects CAMs from oxidative damage. Activation of de novo lipogenesis in cancer cells leads to increased membrane lipid saturation, resulting in higher levels of saturated and monounsaturated phospholipids, which may protect cancer cells from free radicals and chemotherapeutics [25].

Transcriptional analysis of the unique gene set identified in hypoxic NTMs shows a more classic response, mainly composed of genes known to contribute to hypoxic signalling pathways, hypoxia response, and xenobiotic metabolism.

The IPA downstream effect analysis performed in this study predicts distinct biological effects of hypoxia among different gastric myofibroblast populations. Significantly, activation of autophagy emerged as a key CAM-specific process that was enhanced in response to hypoxia, whereas processes such as organisation of organelle, growth of microtubules, and aneuploidy of cells appeared to be activated only in hypoxic ATMs, while expression and transcription of RNA were the most significantly activated processes in hypoxic NTMs. Interestingly, processes such as mitosis, angiogenesis, invasion of cells, growth of axons, neoplasia of tumour cell lines, and glycolysis of cells were all predicted to be most active in hypoxic NTMs.

Secretome analysis confirmed some of the predictions based on hypoxia-induced CAM and NTM gene expression profiles. Interestingly, lysyl oxidase (LOX), among others, was verified at the protein level as an NTM unique hypoxia-induced secreted protein. LOX is an extracellular amine oxidase that post-translationally modifies collagens and elastin in the extracellular matrix, catalysing the covalent crosslinking of fibres [26], thus contributing to hypoxia-induced metastasis. LOX is highly expressed in hypoxic tumour cells, including breast cancer, where it promotes extracellular matrix remodelling in the lungs, leading to the formation of a metastatic niche [27]. Comparative analysis of secretomes and gene expression profiles revealed that both expression and secretion of LOX is increased in CAMs compared to NTMs under normal conditions. However, when oxygen is limited, LOX is also induced in NTMs, both at the mRNA and protein level.

Comparative profiling of the secretomes of hypoxic CAMs, compared to control CAMs or hypoxic and control NTMs, shows that hypoxia has a significantly greater impact on protein secretion in NTMs than CAMs. This observation is also consistent with IPA downstream effect analysis, which predicts that expression and transcription of RNA is the most significantly activated processes in hypoxic NTMs. Taken together, these data suggest that CAMs are somehow less responsive to low oxygen levels then NTMs.

Proteins differentially secreted by NTMs in response to hypoxia were predicted to be regulated by HIF-1α activation and are known to enhance tumour cell proliferation, invasion, cell movement, angiogenesis, vasculogenesis, and the migration of endothelial cells. Significantly, these data are consistent with experimental data presented in this study, showing increased cancer cell migration and proliferation in response to NTM-hypoxic-CM compared to NTM-ctrl-CM. By contrast, proteins differentially secreted by hypoxic CAMs, compared to control CAMs, were predicted to reduce cell death via necrosis or apoptosis. Taken together, these results concur with gene expression analysis of hypoxia-induced NTM- and CAM-specific gene signatures.

In summary, this study is the first to perform comparative multi-omics profiling to investigate the global effects of hypoxia on DNA methylation, gene expression, and protein secretion in populations of primary gastric myofibroblasts, derived from either cancer (CAMs), pre-neoplastic (ATMs), or normal (NTMs) tissue. Data presented in this study provide new insight into the differential molecular responses of myofibroblasts to hypoxic conditions within the gastric tumour microenvironment. The integrated multi-omic data sets from this work will act as a resource to inform future studies into the molecular mechanisms of tumour progression, while also facilitating comparative analysis of the effects of hypoxia on myofibroblast function in the microenvironment of other solid tumours.

## 4. Materials and Methods

### 4.1. Primary Gastric Myofibroblasts

Human primary gastric myofibroblasts were derived as previously described [28]. In each case, isolated myofibroblast populations were analysed to confirm stellate/spindle-shaped morphology and consistent expression of both α-SMA and vimentin, as shown previously [5]. Primary myofibroblast cultures were maintained in Dulbecco’s modified Eagle’s medium (DMEM) supplemented with 10% foetal bovine serum (FBS), 1% penicillin–streptomycin, 1% antibiotic–antimycotic, and 1% non-essential amino acid solution. Medium was replaced every 48–60 h. In all cases, myofibroblasts were not maintained beyond passage 12.

### 4.2. Gastric Cancer Cell Culture

Human gastric adenocarcinoma cells (AGS) were cultured in Dulbecco’s modified Eagle’s medium (DMEM) supplemented with 10% foetal bovine serum (FBS), 1% penicillin–streptomycin, and 1% antibiotic–antimitotic solution. The AGS cells were incubated at 37 °C in a humidified atmosphere with 5% CO_2_ and were passaged or growth media were changed approximately every 48–72 h. In all experiments, cells were used between passages 18 and 28.

### 4.3. Hypoxic Conditioned Media Preparation

In order to generate CAM or NTM hypoxic conditioned media (CAM-hypoxic-CM or NTM-hypoxic-CM) and CAM or NTM normoxic conditioned media (CAM-ctrl-CM or NTM-ctrl-CM), 5 × 10^5^ of selected myofibroblast cell lines were seeded in 75 cm^2^ flasks and left attached for 24 h in growth media. The next day, the media were replaced, and cells were put either to normoxia (21% O_2_) or hypoxia (1% O_2_) for 48 h incubation in serum-supplemented DMEM. After 48 h, the cells were washed three times in 1× PBS to get rid of any serum-derived factors, and 13 mL freshly prepared serum-free DMEM supplemented with 1% penicillin–streptomycin, 1% antibiotic–antimycotic, and 1% non-essential amino acid solution was added to the cells. The serum-free media were incubated with the cells for 24 h in normal (21% O_2_) and hypoxic (1% O_2_) conditions. The following day, myofibroblast ctrl-CM and hypoxic-CM were collected and centrifuged at 800*g* for 7 min to remove cell debris. The freshly prepared CAM or NTM hypoxic-CM and ctrl-CM were immediately used for cancer cell migration and proliferation assays.

### 4.4. Cancer Cell Migration Assay

The effects of hypoxic-CAM and hypoxic-NTM conditioned media (CM) on gastric cancer cells migration were measured in vitro using trans-well Boyden chamber assay (SLS; cat. no. 354578). Briefly, 1 × 10^4^ AGS cells in 500 μL of serum-free DMEM medium were added to the BioCoat Control Inserts with 8μm pore PET membrane (upper chambers). The lower chambers contained either 750 μL serum-free media, or myofibroblast CM to serve as a chemoattractant. Cells were incubated at 37 °C and allowed to migrate overnight. Thereafter, AGS cells were removed from the upper surface of the membrane by scrubbing with cotton swabs. Cells migrating through the membrane were fixed and detected on the lower surface using Reastain Quick-Diff Kit (Reagena; cat. no. 102164, Toivala, Finland) and then examined under a bright-field microscope. Values for cancer cell migration were obtained by counting fifteen fields per membrane (20× objective) and represent an average of at least three independent membranes.

### 4.5. Cancer Cell Proliferation Assay

The effects of hypoxic-CAM and hypoxic-NTM conditioned media (CM) on gastric cancer cell proliferation were assessed by incorporation of 5-ethynyl-2′-deoxyuridine (EdU) [29] and detected using the Click-iT EdU Alexa Fluor 488 Imaging Kit (Life Technologies; cat. no. C10337, California, CA, USA). Briefly, 1 × 10^4^ AGS cells were plated onto cover glasses in 24-well plate and left for 24 h at 37 °C to attach. The next day, growth media were replaced with serum-free DMEM supplemented with 1% penicillin–streptomycin and 1% antibiotic–antimycotic to synchronise the cells. Thereafter, the media were replaced with 1 ml of either serum-free media or myofibroblast CM containing 10 μM EdU, and cells were incubated overnight at 37 °C, 5% CO_2_. Following incubation, the manufacturer’s protocol was applied to fix and permeabilise the AGS cells and detect EdU incorporation.

### 4.6. Integrated Multi-Omics Myofibroblast Profiling

Gastric cancer patient-matched CAMs and ATMs (*n* = 3) as well as unrelated NTMs (*n* = 3) were cultured in parallel for integrated genome-wide DNA methylation, gene expression, and secretome profiling. In order to generate myofibroblast conditioned media, 500 × 10^3^ of selected myofibroblast cell lines were seeded in 75 cm^2^ flasks. Growth media were replaced after 24 h and cells were incubated under normoxic (21% O_2_) or hypoxic (1% O_2_) conditions for 48 h. After this initial incubation, cells were washed three times in 1× PBS to remove serum-derived proteins, and 13 mL of freshly prepared serum-free DMEM supplemented with 1% penicillin–streptomycin, 1% antibiotic–antimycotic, and 1% non-essential amino acid solution was added and conditioned for 24 h under normoxic or hypoxic conditions. Conditioned media and cell lysates for DNA and RNA extraction were collected for use in Illumina Infinium HumanMethylation450k and HumanHT-12v4 Expression arrays as well as LC-MS/MS secretome analysis.

#### 4.6.1. Illumina Human HT-12 v4 Array

Total RNA for gene expression analysis using the Illumina HumanHT-12v4 Expression BeadChip arrays was purified using miRNeasy Mini Kit (Qiagen, cat. no. 217004, Hilden, Germany) according to manufacturer’s instructions. RNA sample quality and quantity were assessed by spectrophotometry at 260/280 nm. The Bioconductor package lumi (version 2.18.0) [30] was used to import the raw intensities data into R and perform background correction, variance stabilisation transformation [31], robust spline normalisation, and subsequent quality control. A biological replicate of one of the samples was included to assess the reproducibility of sample preparation (*R^2^* = 0.9933573). Probes with a detection *p*-value < 0.01 across all samples were considered non-detectable and were removed from analysis. Un-annotated probes were also removed, restricting the subsequent analysis to 18090 probes (corresponding to 13381 genes; GEO accession number: GSE125177). To identify differentially expressed transcripts in (i) CAM hypoxia vs. CAM normoxia, (ii) NTM hypoxia vs. NTM normoxia, and (iii) ATM hypoxia vs. ATM normoxia comparisons, the Bioconductor package limma [32,33] was used. The Benjamini and Hochberg method [34] was used to control for false discovery rate.

#### 4.6.2. Illumina Infinium HumanMethylation450k Array

Genomic DNA for DNA methylation analysis using the Illumina Infinium HumanMethylation450 BeadChip arrays was purified using a standard phenol/chloroform extraction method. DNA sample purity and extent of degradation were determined by spectrophotometry at 260/280 nm and gel electrophoresis on 1% agarose gel, respectively. DNA quantity was assessed using PicoGreen fluorimetry (Life Technologies, cat. no. Q-33130, California, CA, USA). Raw DNA methylation data were processed and analysed using Bioconductor package RnBeads version 0.99.17 [35] as described previously [6].

#### 4.6.3. Secretome Profiling

Conditioned media from normoxic and hypoxic myofibroblasts were centrifuged at 800 *g* for 7 min to remove cell debris and supernatants were aliquoted and stored at −80 °C prior to LC-MS/MS secretome analysis, which was performed by the Centre for Proteome Research (University of Liverpool, Liverpool, UK). Raw mass spectrometry data were analysed using MaxQuant software [36] with Andromeda [37] as a search engine against the reference human proteome (Uniprot proteome ID UP000005640, containing 42112 reviewed canonical and isoform protein entries, accessed 30 April 2015) and 123 potential contaminants. Protein quantification was obtained using the MaxQuant intensity-based label free quantification (LFQ) algorithm. Potential contaminants identified in the mass spectrometry data were manually examined, and proteins associated with foetal bovine serum (FBS) or trypsin were rejected. The LFQ algorithm extracts protein intensities form the raw data and performs appropriate normalisation steps to allow between sample comparisons and remove any systematic errors during data acquisition. For simplicity, the protein group was reported in all cases where a protein was identified by peptides that could not be assigned to individual protein unambiguously, for example, in cases of highly homologous proteins, or protein isoforms. On average, each protein was quantified with 7 unique peptides and 22% sequence coverage was achieved. To compare CAM and NTM secretomes from different microenvironmental conditions, four pair-wise comparisons were done using a one-way analysis of variance (ANOVA) test as implemented in DanteR software [38]. Proteins with a *p*-value < 0.05 were considered to be differentially secreted between the two conditions. The raw mass spectrometry secretome data and processing results from MaxQuant have been deposited to the ProteomeXchange Consortium (http://proteomecentral.proteomexchange.org) via the PRIDE partner repository [39] with the identifier PXD008104.

### 4.7. Gene Ontology Enrichment Analysis

Gene Ontology (GO) enrichment analysis [40] was performed on hypoxia-induced gene expression profiles identified in CAMs, ATMs, and NTMs using GOrilla (gene ontology enrichment analysis and visualization tool) [41]. The target genes were compared to the background gene set of all expressed and normalised genes from the HT-12v4 Illumina experiment. The enriched GO terms in individual CAM, ATM, and NTM hypoxia-induced gene expression profiles were discovered using a hypergeometric model, *p*-value < 0.001 was applied, and the results were corrected for multiple testing using the Benjamini and Hochberg method [34]. To reduce redundancy within the identified enriched lists of GO terms, web server REVIGO [42] was used.

### 4.8. Gene Set Enrichment Analysis

Gene set enrichment analysis (GSEA v5.0) [43,44] was used to characterise and interpret the identified unique-CAM, ATM, or NTM and universal hypoxia-induced gene expression profiles. The Molecular Signatures Database (MSigDB v5.0) [44] that contains collection of biologically predefined gene sets was used to determine statistically enriched gene sets present in the analysed gene expression data. The identified unique-CAM, ATM, or NTM and universal hypoxia-induced gene expression profiles were separated into two phenotypes for GSEA: Hypoxia and normoxia. For gene list ranking, multiple probes matching the same gene were sorted according to *p*-value, and the probe with the lowest *p*-value was retained for the analysis. Genes were ranked using the provided signal-to-noise ranking statistic, and GSEA was run using default weighted enrichment statistics and evaluated for statistical significance by comparison to results obtained using 1000 random permutations of each gene set. Default settings were used for all other GSEA parameters.

### 4.9. Ingenuity Pathway Analysis

Ingenuity pathway analysis (IPA) software was used to analyse and interpret the hypoxia-induced gene expression profiles identified in CAMs, ATMs, and NTMs and hypoxia-induced CAM and NTM secretome profiles. Gene expression profiles were compared against an IPA predefined Illumina HT-12v4 reference set, whereas secretome profiles were compared against an Ingenuity Pathway Analysis knowledge base (IKB). For gene expression data, an IPA canonical pathway and downstream effects analyses were performed. For secretome data, IPA downstream effects and upstream regulator analyses were performed. In each case, *p*-value was assigned using Fisher’s exact test, which indicates the probability of overlap between the pathway/phenotype and input genes.

### 4.10. R/Bioconductor

R statistical software (version 3.1.2) and Bioconductor [45,46] were used to process and analyse gene expression, DNA methylation, and secretome data.

## 5. Conclusions

This study shows that patient-derived gastric cancer-associated myofibroblasts (CAMs) exhibit unique changes in gene expression and protein secretion in response to hypoxic conditions. Although hypoxia increased production of factors involved in matrix remodelling in both CAMs and NTMs, upregulation of factors required for cholesterol production and metabolism was only seen in CAMs. These findings suggest that gastric CAMs, unlike patient-matched ATMs or NTMs, contribute to cancer cell growth and metabolism. Functional studies show hypoxia increases the ability of gastric CAMs to enhance cancer cell proliferation and migration, while also conferring pro-tumorigenic properties in non-cancer derived NTMs. Conditional multi-omics data from this study will provide a resource to facilitate future comparative studies to identify common mechanisms of tumour myofibroblast interactions in other solid tumours.

## Supplementary Materials

Available online at https://www.mdpi.com/2072-6694/11/2/263/s1. Figure S1: Differential effects of CAM and NTM hypoxic conditioned media (CM) on AGS gastric cancer cell migration and proliferation, Figure S2: Universal changes induced by hypoxia in gastric CAMs, ATMs and NTMs, Figure S3: GSEA enrichment plots for the most significantly enriched A. hallmark gene set and B. canonical pathway gene set, Table S1: GSEA result summary, Table S2: Gene ontology (GO) biological process (BP) enrichment for unique hypoxia-induced gene expression signatures identified in CAMs, ATMs or NTMs, Figure S4: GSEA enrichment plots for the most significantly enriched hallmark gene sets, Table S3: GSEA result summary, Figure S5: IPA canonical pathways significantly enriched in unique CAM, ATM and NTM hypoxia-induced gene expression profiles, Figure S6: Predicted biological effects of hypoxia on gastric CAMs, ATMs and NTMs, Figure S7: Venn diagram representation of database searches used to classify proteins identified in CAM and NTM conditioned media obtained from normoxia and hypoxia, Figure S8: Comparison of differentially secreted proteins identified in CAM-hypoxic-CM and NTM-hypoxic-CM compared to their respective normoxic-control-CM, Figure S9: Predicted biological effects of the hypoxia-induced CAM and NTM secretomes, Figure S10: Predicted activation of HIF-1α regulates expression of proteins secreted by hypoxic NTMs.

## References

[1] Ferlay J., Soerjomataram I., Dikshit R., Eser S., Mathers C., Rebelo M., Parkin D.M., Forman D., Bray F. (2015). Cancer incidence and mortality worldwide: Sources, methods and major patterns in GLOBOCAN 2012. Int. J. Cancer.

[2] Bhowmick N.A., Neilson E.G., Moses H.L. (2004). Stromal fibroblasts in cancer initiation and progression. Nature.

[3] Allen M., Louise Jones J. (2011). Jekyll and Hyde: The role of the microenvironment on the progression of cancer. J. Pathol..

[4] Balabanova S., Holmberg C., Steele I., Ebrahimi B., Rainbow L., Burdyga T., McCaig C., Tiszlavicz L., Lertkowit N., Giger O.T. (2014). The neuroendocrine phenotype of gastric myofibroblasts and its loss with cancer progression. Carcinogenesis.

[5] Holmberg C., Quante M., Steele I., Kumar J.D., Balabanova S., Duval C., Czepan M., Rakonczay Z., Tiszlavicz L., Nemeth I. (2012). Release of TGFbetaig-h3 by gastric myofibroblasts slows tumor growth and is decreased with cancer progression. Carcinogenesis.

[6] Najgebauer H., Liloglou T., Jithesh P.V., Giger O.T., Varro A., Sanderson C.M. (2019). Integrated omics profiling reveals novel patterns of epigenetic programming in cancer-associated myofibroblasts. Carcinogenesis.

[7] He X.J., Tao H.Q., Hu Z.M., Ma Y.Y., Xu J., Wang H.J., Xia Y.J., Li L., Fei B.Y., Li Y.Q. (2014). Expression of galectin-1 in carcinoma-associated fibroblasts promotes gastric cancer cell invasion through upregulation of integrin β1. Cancer Sci..

[8] Tang D., Gao J., Wang S., Ye N., Chong Y., Huang Y., Wang J., Li B., Yin W., Wang D. (2015). Cancer-associated fibroblasts promote angiogenesis in gastric cancer through galectin-1 expression. Tumour Biol..

[9] Sun C., Fukui H., Hara K., Zhang X., Kitayama Y., Eda H., Tomita T., Oshima T., Kikuchi S., Watari J. (2015). FGF9 from cancer-associated fibroblasts is a possible mediator of invasion and anti-apoptosis of gastric cancer cells. BMC Cancer.

[10] Yang T.S., Yang X.H., Chen X., Wang X.D., Hua J., Zhou D.L., Zhou B., Song Z.S. (2014). MicroRNA-106b in cancer-associated fibroblasts from gastric cancer promotes cell migration and invasion by targeting PTEN. FEBS Lett..

[11] Zhi K., Shen X., Zhang H., Bi J. (2010). Cancer-associated fibroblasts are positively correlated with metastatic potential of human gastric cancers. J. Exp. Clin. Cancer Res..

[12] Hasegawa T., Yashiro M., Nishii T., Matsuoka J., Fuyuhiro Y., Morisaki T., Fukuoka T., Shimizu K., Shimizu T., Miwa A. (2014). Cancer-associated fibroblasts might sustain the stemness of scirrhous gastric cancer cells via transforming growth factor-β signaling. Int. J. Cancer.

[13] Satoyoshi R., Kuriyama S., Aiba N., Yashiro M., Tanaka M. (2014). Asporin activates coordinated invasion of scirrhous gastric cancer and cancer-associated fibroblasts. Oncogene.

[14] Gilkes D.M., Semenza G.L., Wirtz D. (2014). Hypoxia and the extracellular matrix: Drivers of tumour metastasis. Nat. Rev. Cancer.

[15] Dayan F., Mazure N.M., Brahimi-Horn M.C., Pouysségur J. (2008). A dialogue between the hypoxia-inducible factor and the tumor microenvironment. Cancer Microenviron..

[16] Giaccia A.J., Schipani E., Simon M.C. (2010). Role of carcinoma-associated fibroblasts and hypoxia in tumor progression. Diverse Effects of Hypoxia on Tumor Progression.

[17] Comito G., Giannoni E., Di Gennaro P., Segura C.P., Gerlini G., Chiarugi P. (2012). Stromal fibroblasts synergize with hypoxic oxidative stress to enhance melanoma aggressiveness. Cancer Lett..

[18] Semenza G.L. (1998). Hypoxia-inducible factor 1: Master regulator of O-2 homeostasis. Curr. Opin. Genet. Dev..

[19] Wang G.L., Jiang B.H., Rue E.A., Semenza G.L. (1995). Hypoxia-inducible factor-1 is a basic-helix-loop-helix-pas heterodimer regulated by cellular o-2 tension. Proc. Natl. Acad. Sci. USA.

[20] Mi H., Muruganujan A., Thomas P.D. (2013). PANTHER in 2013: Modeling the evolution of gene function, and other gene attributes, in the context of phylogenetic trees. Nucleic Acids Res..

[21] Meinken J., Walker G., Cooper C.R., Min X.J. (2015). MetazSecKB: The human and animal secretome and subcellular proteome knowledgebase. Database.

[22] Naba A., Clauser K.R., Hoersch S., Liu H., Carr S.A., Hynes R.O. (2012). The matrisome: In silico definition and in vivo characterization by proteomics of normal and tumor extracellular matrices. Mol. Cell. Proteom..

[23] Mathivanan S., Fahner C.J., Reid G.E., Simpson R.J. (2012). ExoCarta 2012: Database of exosomal proteins, RNA and lipids. Nucleic Acids Res..

[24] Nieman K.M., Kenny H.A., Penicka C.V., Ladanyi A., Buell-Gutbrod R., Zillhardt M.R., Romero I.L., Carey M.S., Mills G.B., Hotamisligil G.S. (2011). Adipocytes promote ovarian cancer metastasis and provide energy for rapid tumor growth. Nat. Med..

[25] Rysman E., Brusselmans K., Scheys K., Timmermans L., Derua R., Munck S., Van Veldhoven P.P., Waltregny D., Daniëls V.W., Machiels J. (2010). De novo lipogenesis protects cancer cells from free radicals and chemotherapeutics by promoting membrane lipid saturation. Cancer Res..

[26] Kagan H.M., Li W. (2003). Lysyl oxidase: Properties, specificity, and biological roles inside and outside of the cell. J. Cell. Biochem..

[27] Erler J.T., Bennewith K.L., Cox T.R., Lang G., Bird D., Koong A., Le Q.T., Giaccia A.J. (2009). Hypoxia-induced lysyl oxidase is a critical mediator of bone marrow cell recruitment to form the premetastatic niche. Cancer Cell.

[28] McCaig C., Duval C., Hemers E., Steele I., Pritchard M., Przemeck S., Dimaline R., Ahmed S., Bodger K., Kerrigan D.D. (2006). The role of matrix metalloproteinase-7 in redefining the gastric microenvironment in response to Helicobacter pylori. Gastroenterology.

[29] Salic A., Mitchison T.J. (2008). A chemical method for fast and sensitive detection of DNA synthesis in vivo. Proc. Natl. Acad. Sci. USA.

[30] Du P., Kibbe W.A., Lin S.M. (2008). lumi: A pipeline for processing Illumina microarray. Bioinformatics.

[31] Lin S.M., Du P., Huber W., Kibbe W.A. (2008). Model-based variance-stabilizing transformation for Illumina microarray data. Nucleic Acids Res..

[32] Smyth G.K., Gentleman R., Huber W., Irizarry R., Dudoit S. (2005). Limma: Linear models for microarray data. Bioinformatics and Computational Biology Solutions Using R and Bioconductor.

[33] Smyth G.K., Michaud J., Scott H.S. (2005). Use of within-array replicate spots for assessing differential expression in microarray experiments. Bioinformatics.

[34] Benjamini Y., Hochberg Y. (1995). Controlling the false discovery rate a practical and powerful approach to multiple testing. J. R. Stat. Soc. Ser. B-Methodol..

[35] Assenov Y., Müller F., Lutsik P., Walter J., Lengauer T., Bock C. (2014). Comprehensive analysis of DNA methylation data with RnBeads. Nat. Methods.

[36] Cox J., Mann M. (2008). MaxQuant enables high peptide identification rates, individualized p.p.b.-range mass accuracies and proteome-wide protein quantification. Nat. Biotechnol..

[37] Cox J., Neuhauser N., Michalski A., Scheltema R.A., Olsen J.V., Mann M. (2011). Andromeda: A peptide search engine integrated into the maxquant environment. J. Proteome Res..

[38] Taverner T., Karpievitch Y.V., Polpitiya A.D., Brown J.N., Dabney A.R., Anderson G.A., Smith R.D. (2012). DanteR: An extensible R-based tool for quantitative analysis of -omics data. Bioinformatics.

[39] Jarnuczak A.F., Vizcaíno J.A. (2017). Using the PRIDE Database and ProteomeXchange for Submitting and Accessing Public Proteomics Datasets. Curr. Protoc. Bioinformatics.

[40] Khatri P., Drăghici S. (2005). Ontological analysis of gene expression data: Current tools, limitations, and open problems. Bioinformatics (Oxford, England).

[41] Eden E., Navon R., Steinfeld I., Lipson D., Yakhini Z. (2009). GOrilla: A tool for discovery and visualization of enriched GO terms in ranked gene lists. BMC Bioinform..

[42] Supek F., Bošnjak M., Škunca N., Šmuc T. (2011). REVIGO summarizes and visualizes long lists of gene ontology terms. PLoS ONE.

[43] Mootha V.K., Lindgren C.M., Eriksson K.F., Subramanian A., Sihag S., Lehar J., Puigserver P., Carlsson E., Ridderstråle M., Laurila E. (2003). PGC-1alpha-responsive genes involved in oxidative phosphorylation are coordinately downregulated in human diabetes. Nat. Genet..

[44] Subramanian A., Tamayo P., Mootha V.K., Mukherjee S., Ebert B.L., Gillette M.A., Paulovich A., Pomeroy S.L., Golub T.R., Lander E.S. (2005). Gene set enrichment analysis: A knowledge-based approach for interpreting genome-wide expression profiles. Proc. Natl. Acad. Sci. USA.

[45] Gentleman R.C., Carey V.J., Bates D.M., Bolstad B., Dettling M., Dudoit S., Ellis B., Gautier L., Ge Y., Gentry J. (2004). Bioconductor: Open software development for computational biology and bioinformatics. Genome Biol..

[46] Huber W., Carey V.J., Gentleman R., Anders S., Carlson M., Carvalho B.S., Bravo H.C., Davis S., Gatto L., Girke T. (2015). Orchestrating high-throughput genomic analysis with Bioconductor. Nat. Methods.

