# Supplementary material for: Integrative Omic Profiling Reveals Unique Hypoxia Induced Signatures in Gastric Cancer Associated Myofibroblasts
Source: Cancers (Basel). 2019 Feb 23;11(2):263. doi: 10.3390/cancers11020263 (PMC6406696; doi:10.3390/cancers11020263)
Supplement: Supplementary file 1 [file cancers-11-00263-s001.pdf]

Supplementary Materials: Integrative Omic Profiling Reveals Unique Hypoxia Induced Signatures in Gastric Cancer Associated Myofibroblasts

Hanna Najgebauer, Andrew F. Jarnuczak, Andrea Varro and Christopher M. Sanderson

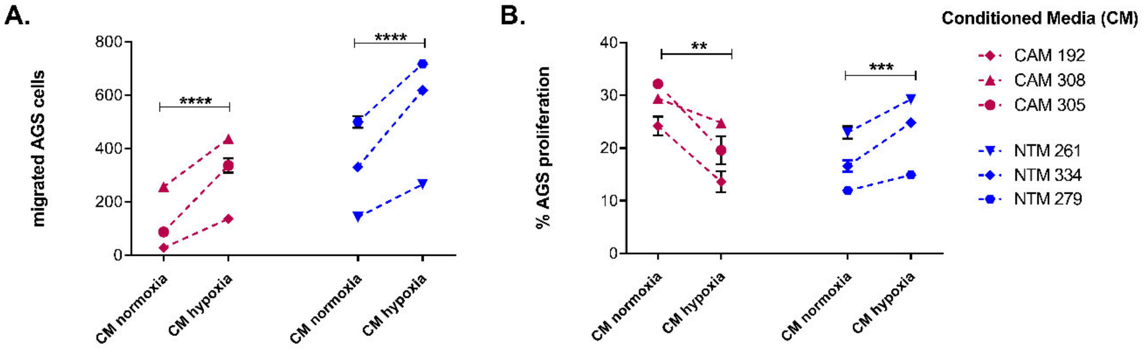

Figure S1. Differential effects of CAM and NTM hypoxic conditioned media (CM) on AGS gastric cancer cell migration and proliferation. **A.** Comparison of AGS cell migration in response to control and hypoxic conditioned media derived from CAMs (n = 3; magenta) and NTMs (n = 3; blue). **B.** Comparison of AGS cell proliferation in response to control and hypoxic conditioned media derived from CAMs (n = 3; magenta) and NTMs (n = 3; blue). Each data point was corrected for AGS basal migration (serum-free media). Error bars represent SEM of technical replicates; paired t-test **** p-value < 0.0001; *** p-value = 0.0001, ** p-value < 0.005.

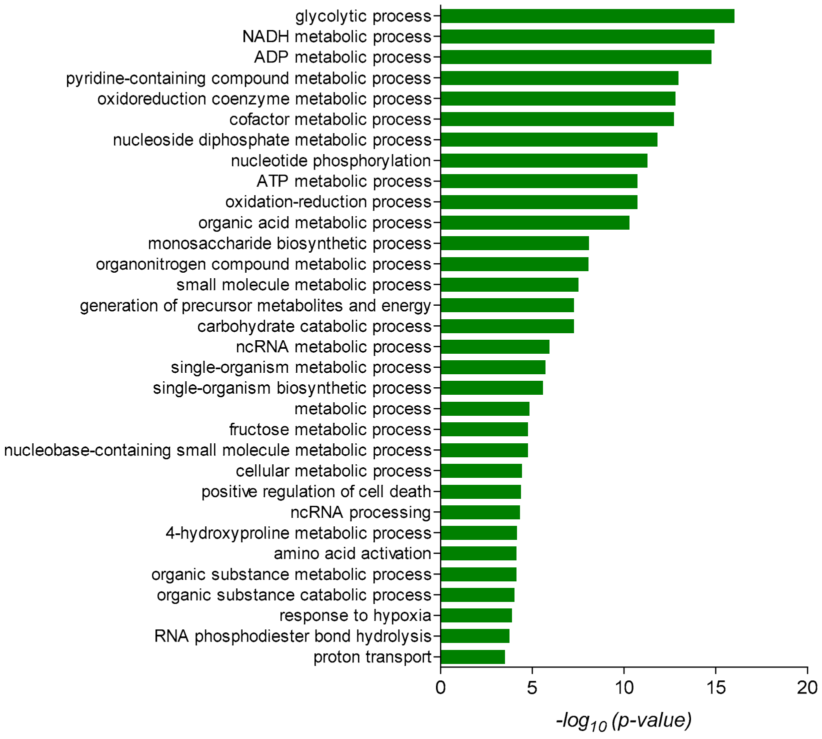

Figure S2. Universal changes induced by hypoxia in gastric CAMs, ATMs and NTMs. Gene ontology (GO) biological process (BP) terms associated with genes that are universally changed under hypoxia in the same direction in CAMs, ATMs and NTMs. GO terms with *FDR* p-value <*0.05* are shown.

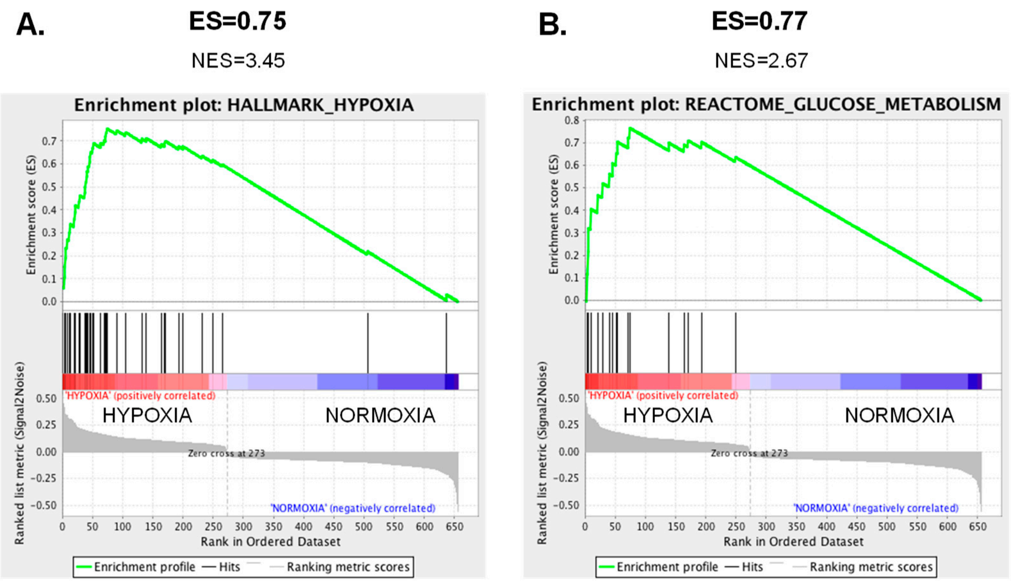

Figure S3. GSEA enrichment plots for the most significantly enriched **A.** hallmark gene set and **B.** canonical pathway gene set. Associated data is presented in Table S1. ES—Enrichment Score, NES—Normalized Enrichment Score.

Table S1. GSEA result summary. Significantly enriched hallmark gene sets (h.all) and canonical pathways (c2.cp) are listed for universal CAM, ATM and NTM gene expression profiles, *FDR p*-value < 0.05. ES—Enrichment Score, NES—Normalized Enrichment Score.

	Phenotype	GENE SET NAME	SIZE	ES	NES	*p*-Value	FDR
h.all	Hypoxia	HALLMARK_HYPOXIA	43	0.75	3.45	0	0
	Hypoxia	HALLMARK_GLYCOLYSIS	35	0.62	2.72	0	0
	Normoxia	HALLMARK_UNFOLDED_PROTEIN_RESPONSE	17	−0.55	−2.04	4.65×10^{-3}	7.75×10^{-3}
	Normoxia	HALLMARK_P53_PATHWAY	21	−0.46	−1.86	4.91×10^{-3}	1.28×10^{-2}
	Hypoxia	HALLMARK_MTORC1_SIGNALING	44	0.38	1.78	6.70×10^{-3}	2.27×10^{-2}
	Hypoxia	HALLMARK_IL2_STAT5_SIGNALING	23	0.45	1.69	1.43×10^{-2}	3.23×10^{-2}
c2.cp	Hypoxia	REACTOME_GLUCOSE_METABOLISM	17	0.77	2.67	0	0
	Hypoxia	REACTOME_METABOLISM_OF_CARBOHYDRATES	26	0.65	2.60	0	0
	Hypoxia	KEGG_GLYCOLYSIS_GLUCONEOGENESIS	19	0.69	2.53	0	0
	Normoxia	REACTOME_METABOLISM_OF_AMINO_ACIDS_AND_DERIVATIVES	17	−0.55	−2.06	2.43×10^{-3}	2.95×10^{-3}
	Hypoxia	NABA_MATRISOME	18	0.54	1.91	3.58×10^{-3}	1.06×10^{-2}

Table S2. Gene ontology (GO) biological process (BP) enrichment for unique hypoxia-induced gene expression signatures identified in CAMs, ATMs or NTMs. GO terms with *FDR p*-value < 0.05 are highlighted in bold.

Myofibroblast Type	GO ID	GO Biologicl Process (BP) Term	*p*-Value
**CAM**	**GO:0006695**	**cholesterol biosynthetic process**	**1.40×10^{-11}**
	**GO:0008299**	**isoprenoid biosynthetic process**	**1.85×10^{-5}**
	GO:0048679	regulation of axon regeneration	2.90×10^{-4}
	GO:0060011	Sertoli cell proliferation	4.88×10^{-4}
	GO:0009448	gamma-aminobutyric acid metabolic process	4.88×10^{-4}
	GO:0008202	steroid metabolic process	6.62×10^{-4}
**ATM**	**GO:0044764**	**multi-organism cellular process**	**1.16×10^{-5}**
	GO:0044267	cellular protein metabolic process	6.71×10^{-5}
	GO:0071360	cellular response to exogenous dsRNA	6.38×10^{-5}
	GO:0043412	macromolecule modification	8.45×10^{-5}
	GO:0016032	viral process	8.78×10^{-6}
	GO:0006464	cellular protein modification process	5.40×10^{-5}
	GO:0043122	regulation of I-kappaB kinase/NF-kappaB signalling	1.22×10^{-4}
	GO:0019538	protein metabolic process	1.18×10^{-4}
	GO:0044419	interspecies interaction between organisms	5.14×10^{-5}
	GO:0010324	membrane invagination	1.45×10^{-4}
	GO:0044260	cellular macromolecule metabolic process	4.51×10^{-5}
	GO:0051704	multi-organism process	1.83×10^{-4}
	GO:1903047	mitotic cell cycle process	2.54×10^{-4}
	GO:0051017	actin filament bundle assembly	3.13×10^{-4}
	GO:0040029	regulation of gene expression, epigenetic	4.09×10^{-4}
	GO:0010660	regulation of muscle cell apoptotic process	4.47×10^{-4}
	GO:0045087	innate immune response	5.07×10^{-4}
	GO:0030522	intracellular receptor signalling pathway	6.80×10^{-4}
	GO:0042059	negative regulation of epidermal growth factor receptor signalling pathway	9.07×10^{-4}
	GO:2000653	regulation of genetic imprinting	9.99×10^{-4}
	GO:0050851	antigen receptor-mediated signalling pathway	9.73×10^{-4}
	GO:0043170	macromolecule metabolic process	9.03×10^{-4}
**NTM**	**GO:0034641**	**cellular nitrogen compound metabolic process**	**3.94×10^{-6}**
	**GO:0006807**	**nitrogen compound metabolic process**	**5.59×10^{-6}**
	GO:0006139	nucleobase-containing compound metabolic process	2.93×10^{-5}
	GO:1901137	carbohydrate derivative biosynthetic process	8.49×10^{-5}
	GO:0090304	nucleic acid metabolic process	8.57×10^{-5}
	GO:0046483	heterocycle metabolic process	8.92×10^{-5}
	GO:0051252	regulation of RNA metabolic process	1.14×10^{-4}
	GO:0009058	biosynthetic process	1.20×10^{-4}

GO:0010648	negative regulation of cell communication	1.29×10^{-4}
GO:0009968	negative regulation of signal transduction	1.48×10^{-4}
GO:0006725	cellular aromatic compound metabolic process	1.51×10^{-4}
GO:1901576	organic substance biosynthetic process	2.77×10^{-4}
GO:1901360	organic cyclic compound metabolic process	3.09×10^{-4}
GO:0051171	regulation of nitrogen compound metabolic process	3.28×10^{-4}
GO:0048585	negative regulation of response to stimulus	3.39×10^{-4}
GO:0045786	negative regulation of cell cycle	3.76×10^{-4}
GO:0023057	negative regulation of signalling	4.11×10^{-4}
GO:0006750	glutathione biosynthetic process	4.34×10^{-4}
GO:0006575	cellular modified amino acid metabolic process	6.32×10^{-4}
GO:0044260	cellular macromolecule metabolic process	6.78×10^{-4}
GO:0006309	apoptotic DNA fragmentation	7.41×10^{-4}
GO:0035878	nail development	8.88×10^{-4}
GO:0044238	primary metabolic process	9.40×10^{-4}
GO:0006488	dolichol-linked oligosaccharide biosynthetic process	9.55×10^{-4}

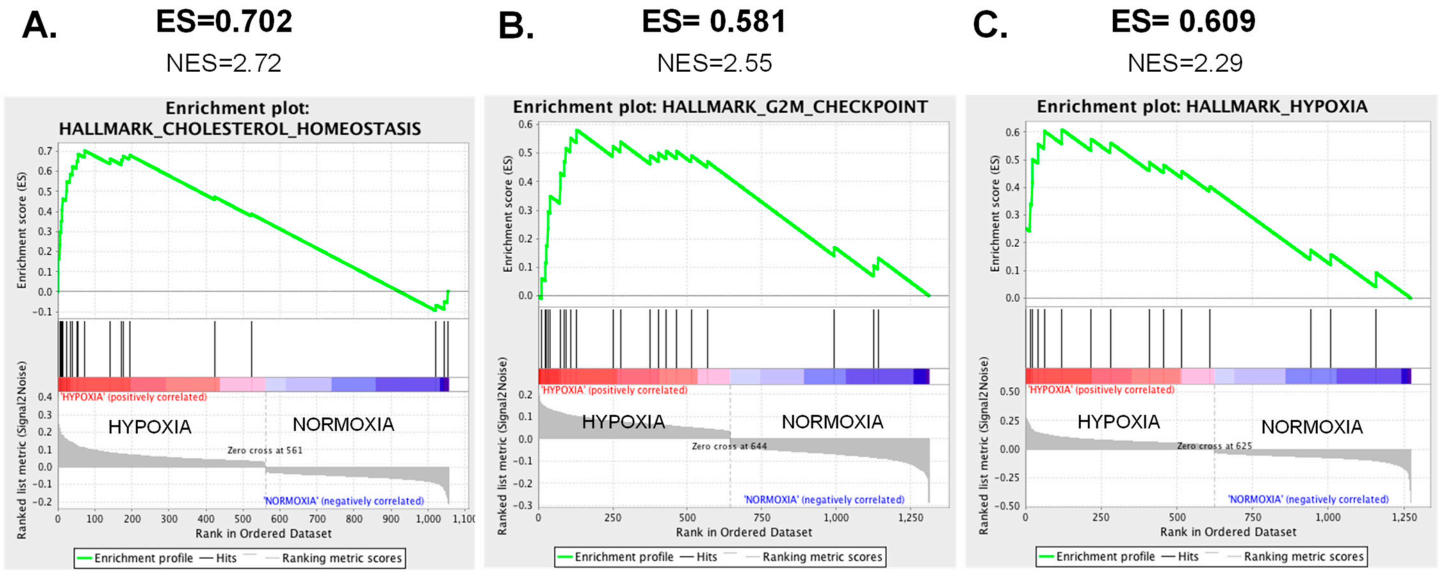

Figure S4. GSEA enrichment plots for the most significantly enriched hallmark gene sets. The most significant hallmark gene set for **A.** CAM unique hypoxia-induced gene expression profile; **B.** ATM unique hypoxia-induced gene expression profile; **C.** NTM unique hypoxia-induced gene expression profile. Associated data is presented in Supplementary Table S3. ES—Enrichment Score, NES—Normalized Enrichment Score.

Table S3. GSEA result summary. Significantly enriched hallmark gene sets are listed for CAM, ATM and NTM unique hypoxia-induced gene expression profiles, FDR p-value < 0.05. ES—Enrichment Score, NES—Normalized Enrichment Score.

Myofibroblast Type	Phenotype	HALLMARK GENE SET NAME	SIZE	ES	NES	p-Value	FDR
CAM	Hypoxia	HALLMARK_CHOLESTEROL_HOMEOSTASIS	23	0.70	2.72	0	0
	Hypoxia	HALLMARK_MTORC1_SIGNALING	25	0.61	2.41	0	0
	Hypoxia	HALLMARK_FATTY_ACID_METABOLISM	19	0.53	1.96	0	5.17×10^{-3}
	Hypoxia	HALLMARK_EPITHELIAL_MESENCHYMAL_TRANSITION	16	0.53	1.90	4.62×10^{-3}	8.79×10^{-3}
	Hypoxia	HALLMARK_E2F_TARGETS	19	0.46	1.71	1.80×10^{-2}	3.36×10^{-2}
ATM	Hypoxia	HALLMARK_G2M_CHECKPOINT	23	0.58	2.55	0	0
	Hypoxia	HALLMARK_EPITHELIAL_MESENCHYMAL_TRANSITION	16	0.62	2.40	0	0
	Hypoxia	HALLMARK_E2F_TARGETS	23	0.46	2.06	0	5.33×10^{-3}
	Hypoxia	HALLMARK_MITOTIC_SPINDLE	24	0.42	1.92	1.14×10^{-2}	1.49×10^{-2}
	Hypoxia	HALLMARK_APICAL_JUNCTION	26	0.38	1.73	1.62×10^{-2}	4.15×10^{-2}
NTM	Hypoxia	HALLMARK_HYPOXIA	16	0.61	2.29	0	0
	Normoxia	HALLMARK_XENOBIOTIC_METABOLISM	28	−0.54	−2.49	0	0
	Normoxia	HALLMARK_UNFOLDED_PROTEIN_RESPONSE	18	−0.61	−2.32	0	0
	Hypoxia	HALLMARK_MITOTIC_SPINDLE	17	0.58	2.18	0	1.84×10^{-3}
	Hypoxia	HALLMARK_EPITHELIAL_MESENCHYMAL_TRANSITION	21	0.55	2.24	0	2.38×10^{-3}
	Hypoxia	HALLMARK_KRAS_SIGNALING_UP	17	0.49	1.86	3.85×10^{-3}	2.74×10^{-2}

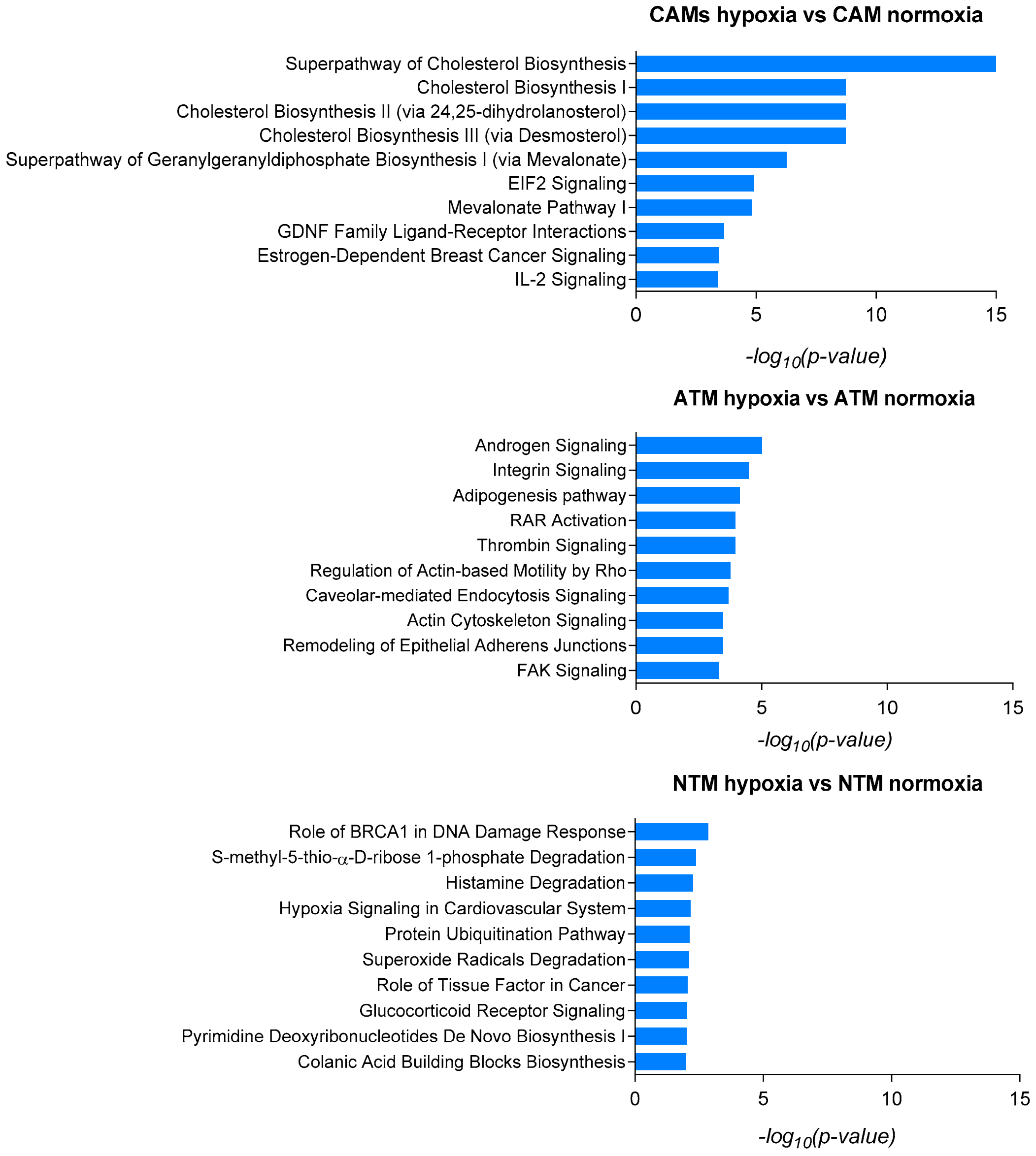

Figure S5. IPA canonical pathways significantly enriched in unique CAM, ATM and NTM hypoxia-induced gene expression profiles. Expression profiles were compared against the IPA predefined Illumina HT-12v4 reference set. Top 10 pathways in respective hypoxia vs normoxia comparisons (indicated at the top of the bar chart) are shown; Fisher's Exact test *p*-value.

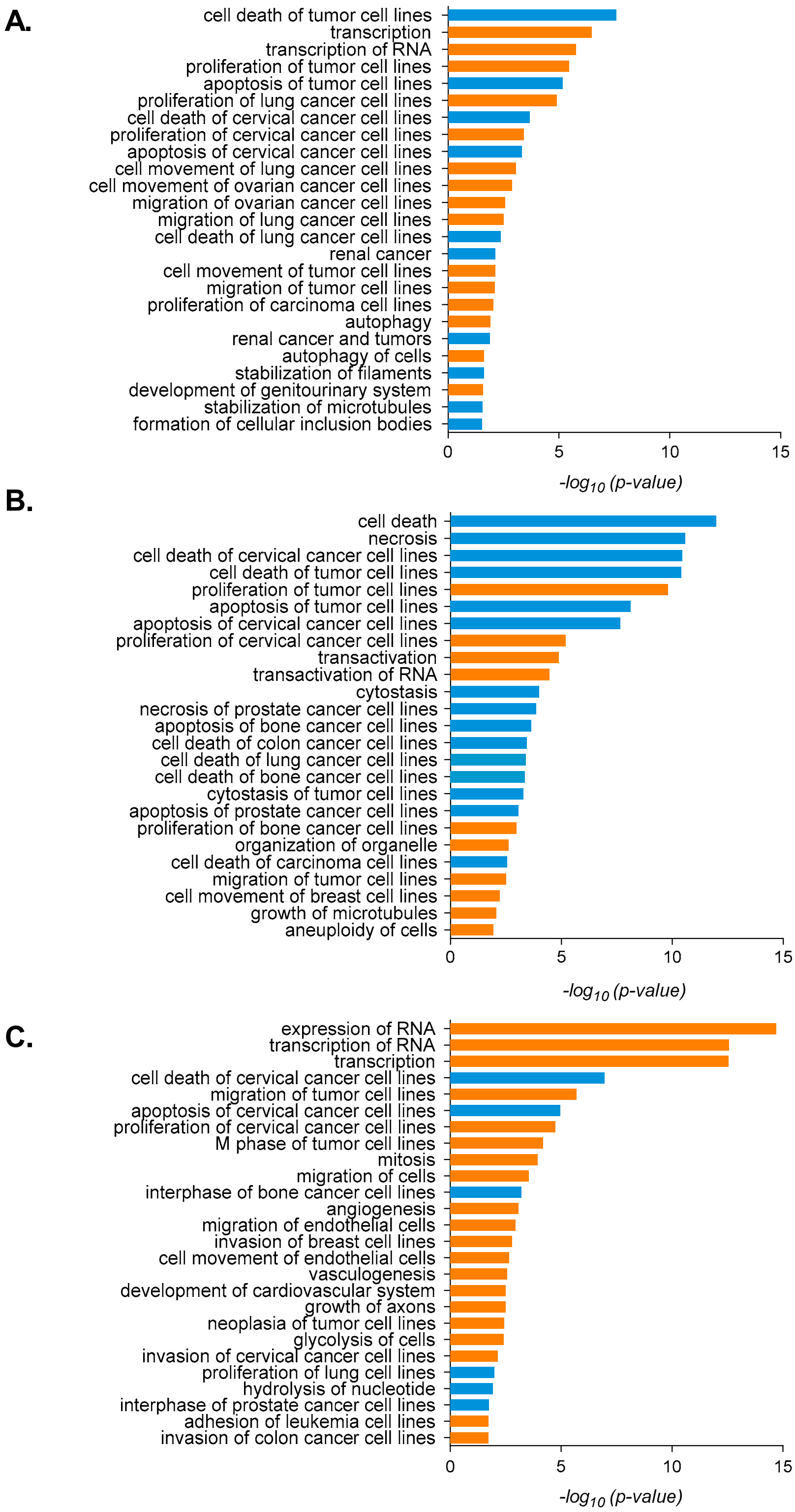

Figure S6. Predicted biological effects of hypoxia on gastric CAMs, ATMs and NTMs. **A.** CAM hypoxia vs. CAM normoxia. **B.** ATM hypoxia vs ATM normoxia. **C.** NTM hypoxia vs NTM normoxia; orange—predicted increase (z-score ≥ 2), blue—predicted decrease (z-score ≤ −2); Fisher's Exact test p-value

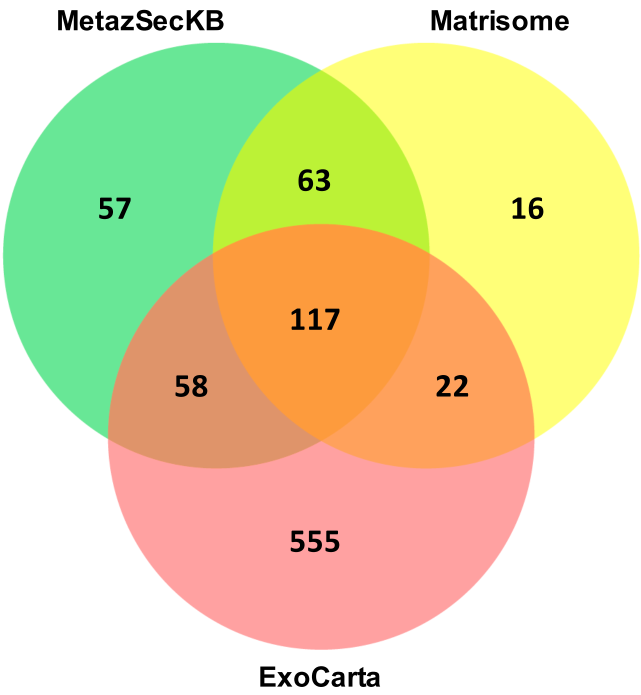

Figure S7. Venn diagram representation of database searches used to classify proteins identified in CAM and NTM conditioned media obtained from normoxia and hypoxia.

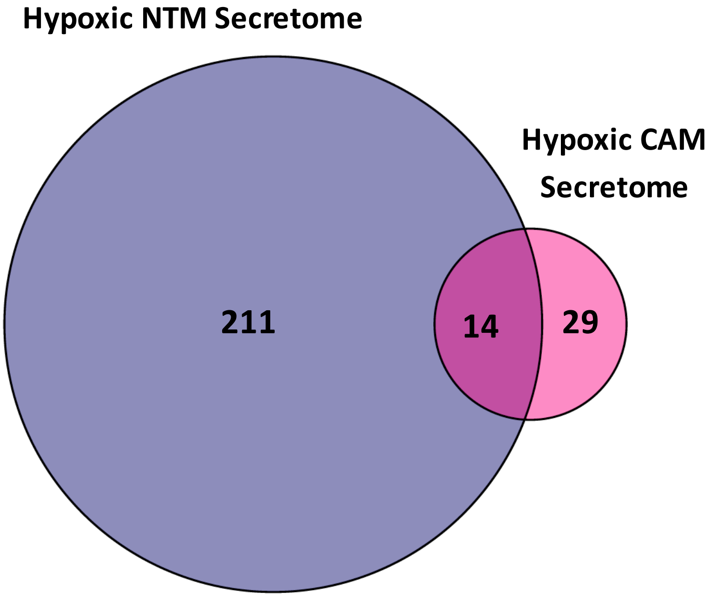

Figure S8. Comparison of differentially secreted proteins identified in CAM-hypoxic-CM and NTM-hypoxic-CM compared to their respective normoxic-control-CM.

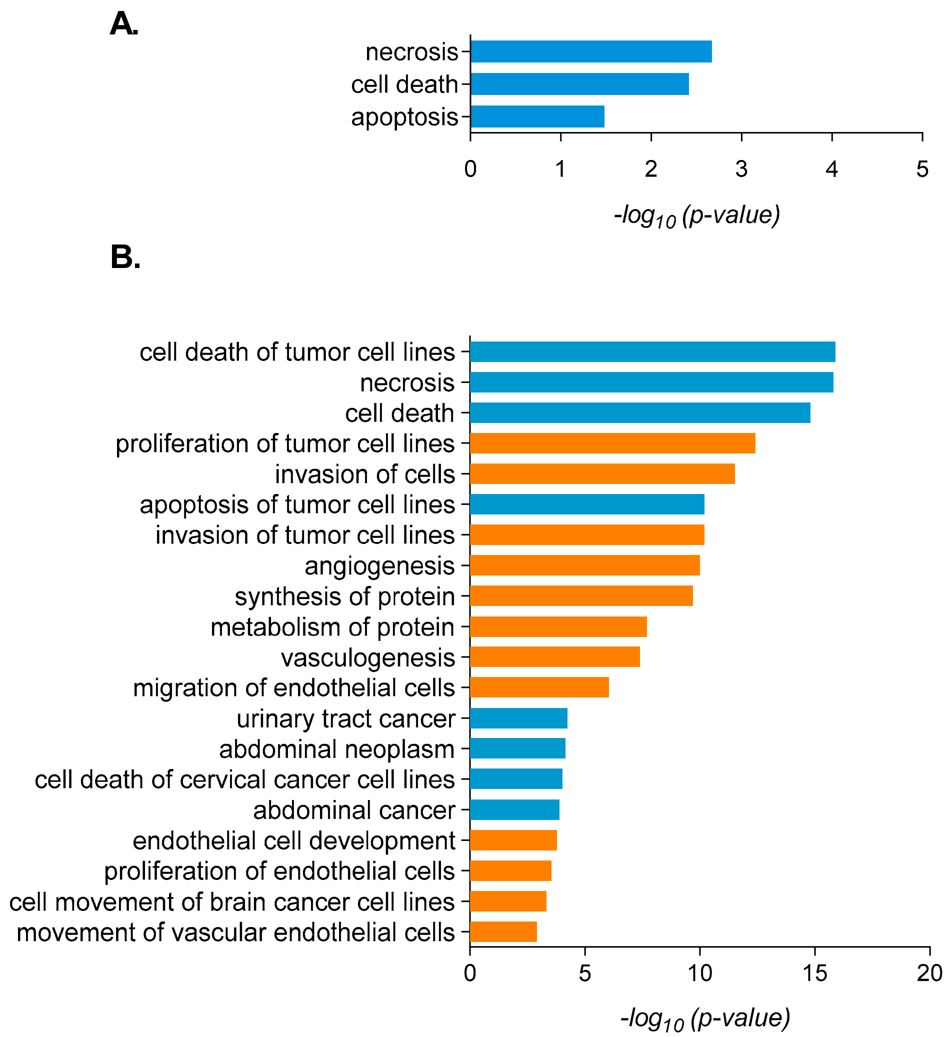

Figure S9. Predicted biological effects of the hypoxia-induced CAM and NTM secretomes. **A.** CAM-hypoxic-CM vs.CAM-ctrl-CM **B.** NTM-hypoxic-CM vs NTM-ctrl-CM; orange—redicted increase (*z*-score ≥ 2), blue—redicted decrease (*z*-score ≤ 2); Fisher's Exact test *p*-value.

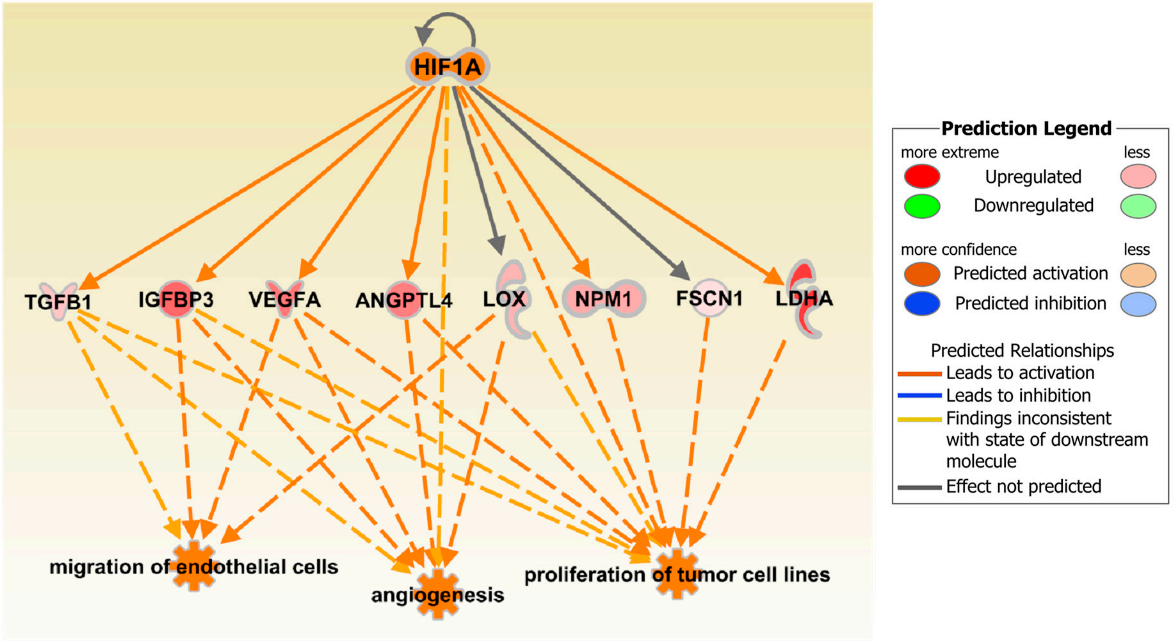

Figure S10. Predicted activation of HIF-1α regulates expression of proteins secreted by hypoxic NTMs. The hypoxic NTM secreted proteins positively affect angiogenesis, migration of endothelial cells and proliferation of tumour cell lines.

© 2019 by the authors. Licensee MDPI, Basel, Switzerland. This article is an open access article distributed under the terms and conditions of the Creative Commons Attribution (CC BY) license (http://creativecommons.org/licenses/by/4.0/).
